# Ectopic Tertiary Lymphoid Tissue in Inflammatory Bowel Disease: Protective or Provocateur?

**DOI:** 10.3389/fimmu.2016.00308

**Published:** 2016-08-16

**Authors:** Eóin N. McNamee, Jesús Rivera-Nieves

**Affiliations:** ^1^Mucosal Inflammation Program, Department of Anesthesiology, School of Medicine, University of Colorado – Anschutz Medical Campus, Aurora, CO, USA; ^2^Division of Gastroenterology, Inflammatory Bowel Disease Center, San Diego VAMC, University of California San Diego, La Jolla, CA, USA

**Keywords:** tertiary lymphoid tissue, ectopic lymphoid follicle, Crohn’s disease, inflammatory bowel disease

## Abstract

Organized lymphoid tissues like the thymus first appeared in jawed vertebrates around 500 million years ago and have evolved to equip the host with a network of specialized sites, strategically located to orchestrate strict immune-surveillance and efficient immune responses autonomously. The gut-associated lymphoid tissues maintain a mostly tolerant environment to dampen our responses to daily dietary and microbial products in the intestine. However, when this homeostasis is perturbed by chronic inflammation, the intestine is able to develop florid organized tertiary lymphoid tissues (TLT), which heralds the onset of regional immune dysregulation. While TLT are a pathologic hallmark of Crohn’s disease (CD), their role in the overall process remains largely enigmatic. A critical question remains; are intestinal TLT generated by the immune infiltrated intestine to modulate immune responses and rebuild tolerance to the microbiota or are they playing a more sinister role by generating dysregulated responses that perpetuate disease? Herein, we discuss the main theories of intestinal TLT neogenesis and focus on the most recent findings that open new perspectives to their role in inflammatory bowel disease.

Quis custodiet ipsos custodes

“Who will guard the guardians?” from Juvenal (Satire VI, lines 347–8).

Intrinsic to the gastrointestinal tract, gut-associated lymphoid tissue (GALT) are the sentinels of the enteric immune system and guard the host from an ever-present microbial and antigenic assault. Cryptopatches (CP), isolated lymphoid follicles (ILF), Peyer patches (PP), and the chains of mesenteric lymph nodes (MLN) respond to microbial and immune signals, allowing for rapid remodeling during infection and disease. However, during periods where chronic inflammation persists in the setting of failed immunoregulation, such as in inflammatory bowel diseases (IBD), a dysfunctional lymphatic system and the development of ectopic tertiary lymphoid tissue (TLT) develop as a consistent pathological hallmark. Understanding the function of TLT and the myriad of cellular events leading to their development is becoming an area of intense clinical interest, as their role in pathophysiology remains enigmatic. A critical question remains unanswered; do TLT develop to protect the vulnerable, immune-compromised intestine or do they play a more sinister role in driving autoimmune processes and perpetuate disease?

## Gut-Associated Lymphoid Tissue: *Gatekeepers in Host Defense*

The GALT is the largest collection of lymphoid tissues in the body, consisting of both organized lymphoid tissues (MLN and PP) and more diffusely scattered lymphocytes in the intestinal lamina propria (LP) and intraepithelial space. With the immunologic maturation of the intestine after birth, aggregates of organized leukocyte populations form CP and ILF and collectively with PP and MLN are a crucial interface between the host and commensal bacteria.

### Prenatal GALT Neogenesis

During lymph node development in embryogenesis, a novel subset of CD4^+^ CD3^−^ cells, was identified to play a crucial initiating role (1). Now termed lymphoid tissue inducer (LTi) cells, these hematopoietic progenitors signal to mesenchymal cell subsets (stromal organizer cells) within the developing lymph node Anlagen [reviewed in Ref. (2)]. Initial signaling *via* stromal-derived LTβR with its ligand, lymphotoxin-α_1_β_2_ (LTα_1_β_2_) on LTi’s, drives a cascade of chemokine and stromal markers, which recruit and organize immune cells into the developing lymphoid tissue (2). LTi are now identified as members of the innate lymphoid cells (ILC) [specifically type 3 ILC (ILC3)], which express the transcription factors, helix–loop–helix protein inhibitor of DNA binding 2 (ID2) and RAR-related orphan receptor gamma^+^ (RORγt^+^), in addition to the cytokines IL-22 and IL-17a.

Lymphoid tissue inducer cells (ILC3) play a particularly crucial role in development of GALT *in utero* [Reviewed in Ref. (3–5)]. For example, MLN develops at embryonic day E11.5, following colonization of the anlagen with LTα_1_β_2_^+^ LTi’s and activation of lymphotoxin-β receptor-expressing (LTβR) stromal organizer cells (6, 7). The importance of this interaction is evident from early murine studies where mice deficient for both RORγt and LTβR lack MLN (4, 8). Interestingly, while distinct regulatory cytokine/chemokine circuits (such as IL-7, LTβ, CXCL13/CXCR5) control MLN function and organization, their absence does not interfere with MLN development (9–12). Of interest, recent work has demonstrated that while LTβR^−/−^ mice fail to develop secondary lymph nodes (SLO), in the setting of excessive TNF production during intestinal inflammation, TNF-α (transgenic over-expression in TNF^ΔARE/+^ mice) over-rides the canonical requirement for LTi cells and drives a lymphoid neogenesis program, including the induction of homeostatic chemokines (13). Thus, subtle differences may still remain between homeostasis and chronic inflammation for the ontogeny and regulation of MLN formation.

Peyer patches, which are scattered along the anti-mesenteric border of the small intestine, drain to the mesenteric lymphatic system *via* efferent lymphatic vessels and directly sample antigen from the gut lumen *via*, specialized microfold cells (M cells) (14, 15). The development of PP in the fetal intestine takes place later than MLN (E11.5) between E.15.5 and E18.5 and is also critically dependent on LTβR signaling and CD11c^+^ dendritic cells (DC) (16, 17). This is most evident by the observation that mice deficient in LTα and LTβ, and as such for their membrane ligand LTα_1_β_2_, lack mature PP (8, 9). Critically, LTβR ligation signals *via* the alternative NFκB pathway to induce CXCL13 and recruit LTi and CXCR5^+^ B cells for PP maturation (6, 18). In addition, while TNF is not required for MLN ontogeny *in utero*, TNF and TNFRI (and signaling *via* classical NFκB pathway) are required for PP development (19, 20).

### Postnatal GALT Neogenesis – Integrating Environmental and Commensal Stimuli

Aside from the developmental program of GALT organogenesis, the mammalian intestine adapts and responds to their postnatal colonization by enteric flora with the induction of CP and ILFs.

Cryptopatches are aggregates of approximately 1000 cells composed of LTi cells and chemokine producing dendritic (DC) and stromal cells found around the crypts of the small intestine (21, 22). In response to commensal bacterial stimuli, CP recruit B cells and CD4 T cells to develop into ILF and play a major regulatory role in the intestine by producing Immunoglobulin A (IgA) (23). ILFs are loosely organized clusters of B cells, DC, and T cells that resemble secondary lymphoid organs (SLO) in their cellular components (24, 25). A series of pioneering studies extended on this observation and demonstrated that CP and ILFs utilize similar pathways to SLO for development, following stimulation by a TNF-Lymphotoxin signaling axis (21–27). Expression of the chemokine receptor CCR6 by B cells is critical for expansion of ILFs. The CCR6 ligand, CCL20 is expressed by the epithelial cells that overlay the B cell follicles and its expression, is regulated by LTα_1_β_2_ signaling (28).

Recent work has broadened our understanding for the role of ILFs, and a general consensus is that they act in a tolerogenic manner to control intestinal immune responses by generating both IgA^+^ plasma cells and regulatory T cells (26, 29, 30). It is now apparent that intestinal ILF form a feedback loop with commensal bacteria, whereby there is reciprocal crosstalk. As an example, the induction of the NOD1 receptor (nucleotide-binding oligomerization domain containing 1) in intestinal epithelial cells by Gram-negative bacteria induces ILFs from CP precursors (31). Conversely and strikingly, in the absence of ILFs (following LTβR-IgG treatment), there is a 10-fold expansion of bacterial flora (31). It is not surprising then that ILFs have been tasked with building postnatal intestinal immune tolerance, *via* generation of IgA and Th17 responses (32). Of note however is that the chain of molecular events required for ectopic lymphoid tissue development under conditions of chronic inflammation and their role in the pathogenesis of CD are less clear.

## Intestinal Tertiary Lymphoid Tissue in Inflammatory Bowel Disease

While SLO is developmentally controlled with fixed anatomic locations, chronic inflammation in peripheral tissues can give rise to the florid development of TLT neogenesis [reviewed in Ref. (33)]. Unlike SLO, TLT do not possess a capsule and as such are not true organs *per se* but rather a highly organized cluster of immune cells, which develop regional segregation similar to SLO. While intestinal ILF are loosely organized clusters of B cells, T cells, and DCs, TLT are defined by the presence of densely packed and active germinal centers (GCs) often with mature follicular dendritic cell (FDC) networks (34). They are further defined by presenting with CD4^+^ T cell and DC clusters in addition to a mature fibroblastic stromal network (e.g., VCAM1^+^). TLT often develop around lymphatic vessels and a hallmark indication of mature TLT is the development of specialized high endothelial venules (HEV) within and around follicles (35, 36). As HEV facilitate recruitment of naive (CD62L^+^) T cells into the T cell cortex of SLO (37), their presence in TLT has raised the possibility that TLT bypass the need for SLO by recruiting and educating naive T cells aberrantly in inflamed peripheral tissues.

### The Inflammatory Bowel Diseases, Crohn’s Disease, and Ulcerative Colitis

Inflammatory bowel diseases are a collective of chronic intestinal pathologies predominantly represented by Crohn’s disease (CD) and ulcerative colitis (UC). There are fundamental differences between the two, being best characterized as immune-mediated rather than autoimmune, as up to date no single autoantibody has been identified. The etiology of IBD remains elusive but involves complex interactions between genetic, environmental, and immunoregulatory factors. Current hypotheses propose that damage to the intestinal mucosa occurs as a result of a dysregulated immune response triggered by microbial antigens (38, 39) that eventually becomes autonomous. The resulting increased leukocytic infiltrate within the intestinal mucosa release a cocktail of enzymes, reactive oxygen species, and cytokines initiating and perpetuating tissue damage and disease. Regarding tissue distribution, UC involves strictly the colon, while CD can involve any segment of the GI tract, from the mouth to the anus, but predominantly the immunologically rich terminal ileum in 2/3 of patients. UC is also a continuous superficial disease, involving predominantly the colonic mucosa, while CD is discontinuous and penetrating, involving all layers of the intestine from the mucosa to the serosa.

The prevailing histopathologic hallmarks of CD during its early investigation were occluded lymphatic vessels, lymphocytic lymphangitis, and inflammatory “T_H_1” granulomas. Significantly, these cardinal signs of chronic disease were found in or around ectopic tertiary lymphoid follicles in the inflamed LP [(40–43); Reviewed in Ref. (44)]. Indeed, the presentation of TLT in patient biopsies appears to be a predominant feature of CD [in a recent study, TLT were present in 22 out of 24 patients assessed by immunohistochemistry (45)]. In addition, the presence of TLT at the base of aphthous ulcers is also the earliest endoscopically evident lesion in CD and their appearance heralds recurrent disease within the neoterminal ileum after ilectomy (46–48). In light of these findings, the functional relevance of intestinal TLT and their impact on the etiology and pathogenesis of CD has remained enigmatic, with limited empirical evidence as to their role.

A critical question that remains unanswered is the origin of TLT and whether they are generated *de novo* within the chronically inflamed intestine or predetermined. While the components of mature intestinal TLT include CD4^+^ T cell clusters, follicular DC, HEV networks, and mature fibroblastic stroma clusters (VCAM1^+^ ICAM1^+^) (34), it is unclear whether they arise *de novo*, specifically during chronic inflammation. This question is also at the root of our understanding the functional role of TLT during chronic intestinal disease. While homeostatic ILFs represent a source of IgA to maintain tolerance to commensal bacteria, the transformation of ILF into mature TLT [as has been suggested (23)] could indicate a final detrimental step in the development of intestinal immune dysregulation and the loss of immune-tolerance. The anatomic location of both ILF and TLT within the normal and inflamed intestine may also shed light on their respective functions. While ILFs contain a dome of epithelia containing M cells, TLT are often present at sites of epithelial barrier loss (aphthous ulcers in CD) and around occluded lymphatic vessels in the LP (44, 45). In addition, as CD presents with transmural inflammatory infiltrates, TLT follicles may also be present in the deeper layers of the intestine including the muscle and surrounding mesenteric adipose tissue (e.g., “creeping fat” that encases inflamed intestine in a subset of patients with CD).

Elegant recent work has demonstrated that on a background of failed anti-microbial immunity (*ROR*γ*t*^−/−^ mice; lacking ILC3 and T_H_17 responses) combined with a loss of epithelial barrier function (DSS-colitis), mice develop an aggressive colitis in addition to florid TLT neogenesis (49). Antibiotic treatment reversed this pathology and TLT development confirming its dependence on a commensal microbial insult. However, it is worth noting that the phenotype of TLT that were generated in *ROR*γ*t*^−/−^ mice contributed to systemic pathology, produced high levels of AID (to facilitate class-switch recombination), and were strikingly attenuated following intravenous immunoglobulin (IVIG) treatment (49). Our work and others has further demonstrated that in the TNF^ΔARE/+^ mouse model of Crohn’s-like ileitis [TNF^ΔARE/+^; a transgenic mouse line with a 69-bp deletion of the 3′UTR for TNF, allowing for overexpression of TNF mRNA (50)], TLT develop during chronic disease and correspond with both a loss of immune tolerance and a prominent dysbiosis of commensal microflora (34, 51, 52). Thus, the presence of TLT during chronic intestinal inflammation clearly heralds a failure of organ-specific immune regulation and the establishment of dysregulated immunity. As such, distinct intestinal TLT may develop based upon the inflammatory environments [tolerogenic CP-ILF induction from commensal bacteria versus inflammation-induced TLT (CP independent); as has been previously postulated (5)].

## Possible Classical and Non-Classical Cues for Intestinal B Cell Follicle Development

A vast literature has demonstrated that the molecular cues and cellular machinery required for secondary lymph node development are also utilized for the generation of ectopic TLT during chronic inflammation. For example, Lymphotoxin-β receptor signaling on LTi cells (LTα_1_β_2_^+^ LTi and LTβR^+^ stromal cells) remains a cardinal requirement for both the generation and organization of SLO. This was elegantly demonstrated by the lack of secondary lymph nodes in the LTα, LTβ, and LTβR-deficient mice (6, 9). In addition, antibody blockade of LTβR signaling *in vivo* dissociates ectopic TLT structures in a multitude of settings using preclinical mouse models of inflammatory diseases (49, 53–55).

**Figure 1:**
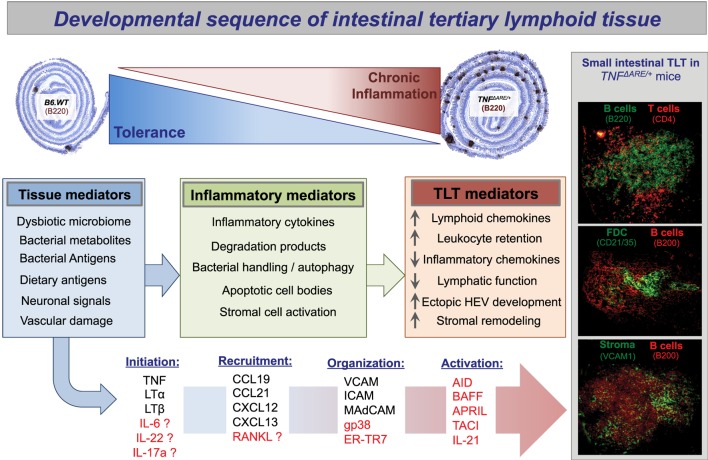
**Propose developmental sequence of intestinal tertiary lymphoid tissue**. Presented here is a proposed sequence of small intestinal tertiary lymphoid tissue neogenesis in the setting of inflammatory bowel disease. On a background of failed immunoregulation and loss of intestinal tolerance, the florid development of ectopic tertiary lymphoid tissue follicles is a cardinal sign of both Crohn’s disease and TNF-transgenic mice with ileitis (TNF^ΔARE/+^). The plasticity of these ectopic lymphoid follicles is demonstrated by the observation that they resolve, for example, after removal of bacterial stimuli by antibiotic treatment or following anti-inflammatory interventions (34, 119). As the etiology of Crohn’s disease is unknown and proposed to be a result of uncontrolled immune activation to intestinal bacteria in genetically susceptible individuals, we have proposed plausible options from the myriad of potential initiating and organizing signals. TLT development is sure to follow a loss of tissue integrity, chronic activation of inflammatory mediators, and stabilization of maturation events to maintain complex TLT architecture. BAFF, B cell activating factor; APRIL, a proliferation inducing ligand; TACI, TNFR homolog transmembrane activator and Ca^2+^ modulator and CAML interactor [APRIL and BAFF receptor]; ER-TR7, anti-reticular fibroblasts and reticular fibroblast marker; gp38, podoplanin; RANKL, receptor activator of nuclear factor kappa-B ligand/TNFSF11.

However, there is also an ever-increasing body of work identifying novel immune pathways that can by-pass the classical sequence for tertiary lymphangiogenesis (depicted in Figure 1). While iILC subsets (to which lymphoid tissue inducer (LTi) cells belong to) are critical for mucosal immunity and for the development of lymph node Anlagen, some reports have identified LTi-redundant mechanisms for TLT development (13, 56, 57). This includes the development of small intestinal TLT driven by TNF-overproduction in the absence of LTi signals (13). TNF production from M_1_-like macrophage also confers an LTi phenotype in stromal cells to generate TLT, independent of LTβR signaling (58). Conversely, in the absence of ILC3 and Th17 anti-microbial responses (*ROR*γ*t*^−/−^) or *LT*α^−/−^, mice develop florid TLT development during colonic inflammatory insults (49, 59). Thus, the cardinal role of the ILC3–Th17 axis in TLT function, during chronic intestinal inflammation and in the heterogeneous and anatomically distinct subsets of IBD, warrants further investigation (Table 1).

**Table 1:** **Incidence of tertiary lymphoid tissue development in human and mouse intestine**.

Condition/model	Location	Lymphoid component(s)	Reference
**Human**			
*Helicobacter Pylori*	Stomach	B/T cells, HEV, CXCL13	(120, 121)
Crohn’s disease	Ileum/Colon	B/T cells, lymphoid follicular inflammation	(44–46, 122)
Ilectomy (Crohn’s disease)	Terminal ileum	Aphthous ulcers, ulcers >8mm, fibrotic strictures rare	(48)
Colorectal carcinoma	Colon	B/T cells, FDC, CXCL13, CCL19, CCL21	(123, 124)
**Mice**			
**Disease model**			
Autoimmune gastritis	Stomach	B/T cells, CXCL13, FDC, autoantibodies	(125, 126)
*Helicobacter Pylori*	Stomach	B/T cells, FDC, GC, CXCR5, CXCL13	(127–129)
TNF-transgenic (*TNF*^ΔARE^)	Ileum	B/T cells, FDC, GC CCL19, CCL21, CXCL13	(13, 34)
Prion disease (*LT*α^−/−^*/LT*β^−/−^)	Small intestine	B cells, FDC, CR1/CR2	(130)
DSS-colitis (*ROR*γ*t*^−/−^)	Colon	B/T cells, Th1, autoantibodies, LTBR-dependent	(49)
DSS-colitis (*LT*α^−/−^)	Colon	B/T cells, CD8	(59)
Anti-LTβR *in utero*	Small intestine	B/T cells, GC, IgA, Th17	(25, 32, 59)
**Genetic model**			
*CCR7*^−/−^	Stomach	B/T cells, FDC, CCL21, CXCL13	(131)
*AID*^−/−^	Small intestine	B/T cells, FDC, hyper IgM	(132)
*CnAalpha*^+/−^	Small intestine	B/T cells, CD11b, TFG-β, IFNγ	(133)
*IL-25*^−^*^/^*^−^	Small intestine	B cells, CD11b, CD11c	(134)
CXCL13-transgenic	Ileum	LTi, IL-22, LTα, LTβ, CXCL13	(65)

### Innate and Adaptive Sources of IL-22

IL-22 is a member of the IL-10 cytokine family that is predominantly expressed by Th17, γδT cells, and ILC3 and plays a critical role in anti-microbial defense at mucosal surfaces (60, 61). Recent work has demonstrated a role for IL-22 in the control of both TLT development and function (62). In a viral-induced model of Sjögrens syndrome, delivery of adenovirus into the salivary glands induced development of TLT that was dependent on IL-22 production, initially from NK1.1^+^ and γδT cells, with expression during chronic disease predominated by classical αβT cells. The authors demonstrate that IL-22 maintained CXCL13 and CXCL12 levels to facilitate B cell clustering. Strikingly, IL-22 blockade resulted in loss of TLT structure in addition to anti-nuclear autoantibody generation (62). The involvement of IL-22 in the function of intestinal TLT has not been formally assessed, but warrants investigation, especially considering the major role played by IL-22 during chronic intestinal inflammation (63, 64). The IL-22–CXCL13 axis may also be a reciprocal one, as over-expression of CXCL13 in the intestine facilitates the recruitment of IL-22^+^ ILCs, B cell clustering, and the generation of ILFs, independent of aberrant inflammation (65). A governing signal that drives both IL-22 and CXCL13 expression is integrated by LTβR, with LTβR initiating an ILC-DC cross talk *via* IL-23 to induce IL-22 following intestinal infection with *Citrobacter rodentium* (66). Surprisingly however, recombinant IL-22 administration can restore TLT formation in the colon of LTβR-deficient mice, suggesting that IL-22 can directly and independently impact TLT organization during intestinal infection (67).

### ILC- and Th17-Derived IL-17a

Other members of the Th17 family, most notably IL-17a, have been implicated in the development of bronchus associated lymphoid tissues (BALT) during lung infections and in a mouse model of Multiple sclerosis (EAE) (68–70). Following infection with *P. aeruginosa*, mice develop extensive BALT formation, which is dependent on IL-17a-driven CXCL12 from the lung stroma (69). In a second study, Rangel-Moreno and colleagues demonstrated that neonatal mice developed BALT following repeated administration of bacterial lipopolysaccharide (LPS), initiated by IL-17-induced CXCL13. Of note, the generation of CXCL13 was also independent of LTβR in this study (68). As the small intestine is the physiologic site for Th17 generation (71, 72), targeted interruption of this cytokine family may reveal a critical role in intestinal TLT function. In line with this, one recent study has demonstrated that segmented filamentous bacteria (SFB) stimulated the postnatal development of ILF and TLT, which substituted for PP as inductive sites for intestinal IgA and SFB-specific T helper 17 (Th17) cell responses (32). How this integrates with chronic intestinal inflammation and the impact of SFB-induced IgA and Th17 from PP and ILF structures during intestinal disease remain to be clarified.

### T Follicular Helper Cells and IL-21

Upon antigen stimulation, naive CD4^+^ helper T cells differentiate into effector subsets with distinctive functions based on the cytokine milieu of their environment (e.g., Th1, Th2, Th17, and T_reg_). A critical function of helper CD4^+^ T cells subsets is to provide stimulatory signals to developing B cells for the generation of appropriate antibody responses. The cardinal CD4^+^ T cell to carry out this function is the T follicular helper cell (Tfh). Tfh localize within lymph node follicles and utilize the chemokine receptor, CXCR5 (receptor for CXCL13) to stimulate and instruct GC reactions leading to Ig class switch and somatic mutation. Tfh perform much of their functions by the generation of the cytokines IL-6 and IL-21 and under the instruction of the transcription factor Bcl-6. An elegant recent study has demonstrated that Th17 cells within PP trans-differentiate into IL-21^+^ Tfh to aid with the development of IgA^+^ plasma cells (73, 74). In the PP, some T_H_17 cells lose their expression of RORγt and IL-17 and convert into Bcl-6^+^ and IL-21^+^ Tfh cells (74). Whether Tfh regulate the GC reactions in ectopic TLT during intestinal inflammation is not well characterized; however, IL-21 is upregulated in the inflamed small intestine of TNF^ΔARE/+^ mice and correlated with the onset of TLT appearance (McNamee and Rivera-Nieves, unpublished observation). IL-21 expression is upregulated in the intestine of patients with IBD and downregulated in anti-TNF responsive CD patients (75, 76). While most current studies have focused on the interplay between IL-21 and Th17 differentiation (77), how IL-21 and Tfh integrate into the organization and function of intestinal TLT have not been assessed.

### Regulatory T Cells

Foxp3^+^ CD4^+^ regulatory T cells (T_reg_) have a unique ability to repress chronic inflammation and are critical for the generation and maintenance of intestinal tolerance and prevention of autoimmunity (78–81). They mediate their suppressive effects by intimately controlling DC activation and by repressing effector T cell proliferation (79, 80, 82). Of note, failed immunoregulation and loss of T_reg_ function is a hallmark of both human IBD and preclinical models (83–85). The first study to demonstrate a link between TLT development and T_reg_ function utilized *CCR7*^−/−^ mice. CCR7 expression is generally high on CD4^+^ Foxp3^+^ T_reg_’s and CD103^+^ regulatory DC. *CCR7*^−/−^ mice have a global loss of these two cell types and inability to control overt inflammation (51). Strikingly, neonatal *CCR7*^−/−^ mice developed BALT without the addition of an extrinsic inflammatory stimulus (86, 87). Importantly, the authors inhibited ectopic BALT follicle development with the adoptive transfer of functional T_reg_ from wild-type mice (87). Thus, the inability of T_reg_ to control chronic intestinal inflammation may facilitate the development and function of TLT during IBD (83–85); however, this has yet to be formally investigated experimentally.

### Follicular Dendritic Cells

In the setting of either IBD or in preclinical models that present with TLT, it has not yet been delineated if lymphoid chemokines from stromal “organizer” cells precede the development of intestinal TLT, or if their activation is dependent on the influx of TNF^+^ and LTα_1_β_2_^+^ leukocytes. This question is of clinical interest as current biologic interventions in IBD are predominantly aimed at depleting lymphocytes, and their affects on stromal compartment in maintaining chronic tissue inflammation are poorly understood. One such cell subset that is critical for active SLO and mature TLT organization, yet understudied, is the FDC. FDC are highly specialized stromal cells, derived from pericytes, arising within active SLO GCs and chronically inflamed tissues to organize TLT (88, 89). FDC form a reticular scaffold for B cells to generate and maintain GC reactions. They possess a unique recycling mechanism to protect captured antigen from degradation and retain it for long-term presentation to B cells with antibody complexes or on complement receptors CR1 and CR2(90). In addition, FDC express CXCL13 and BAFF, which are essential for the recruitment and survival of CXCR5^+^ B cells in GC follicles (91). TLTs require chronic antigenic stimulation for their maintenance and the tonic supply of lymphoid chemokines to conserve their structure. FDCs can perform both of these functions (*via* CR1/CR2 and CXCL13), and their appearance within intestinal TLT heralds the onset of chronic disease and lack of tolerance; however, their source and function during IBD is unknown and warrants investigation. An intriguing question remains whether intestinal FDC can maintain tonic IgA or IgG production from their neighboring B cells.

## Intestinal TLT in IBD and Extraintestinal Disease

### Are Intestinal B Cells Contributing to Immune Dysregulation *via* Generation of Autoreactive or Microflora-Reactive Antibodies?

The classical definition of IBDs (and in particular CD) is that they are “immune-mediated” conditions, triggered by a dysregulated host immune response to commensal microbiome in genetically susceptible individuals and perpetuated by an autonomous or independent dysregulated immune response, which might then become independent of bacterial stimuli. While auto-reactive effector CD4^+^ helper T cell subsets drive a dysregulated immune pathology in CD, neither CD nor UC fall into the category of being classical “autoimmune” conditions, as a pathologic autoantibody has not been identified. However, there is a clear precedent for dysregulated B cells responses in IBD subsets and serological evidence for autoantibodies being generated (92). For example, anti-neutrophil antibodies (ANCA and p-ANCA) are present in patients with UC (60–80%) and to a lesser extent, CD (5–25%) (93–95). Increased concentrations IgG and IgA antibodies to *Saccharomyces cerevisiae* (ASCAs) (brewer’s yeast) are found in 60–70% of patients with CD (96), while IgG antibodies against the *Escherichia coli* outer membrane porin (OmpC) is identified in 55% of CD patients (97). IgG antibodies to the flagellin CBir1 is associated with small-bowel, internal-penetrating, and fibrostenotic disease, and defines a subgroup of CD patients not previously recognized by other serologic responses (92, 98).

Of note, there is now evidence that the magnitude of immune response to different microbial antigens (ASCA and OmpC) in patients with CD is associated with the severity of the disease course (fibrostenosis, internal perforating disease, and the need for small-bowel surgery) (99). Thus, a loss of immune tolerance and generation of autoreactive B cell responses are clear clinical features of CD. Whether this process takes place within the intestinal (and TLT follicles) or is a peripheral response (e.g., spleen and bone marrow) is yet to be determined.

### Are Intestinal TLT a Mucosal Source for the Generation of Extraintestinal Disease during IBD?

A clinical hallmark of IBD is the development of extraintestinal manifestations during its disease course. These include inflammation of the joints, skin, eyes, and hepatobiliary tract, which are the most usually affected sites (100). An interesting observation is that TLT are a predominant feature of CD pathologies, and patients with CD are more likely than UC patients to have immune-mediated (arthritis, eye, skin, and liver) extraintestinal manifestations: 20.1% CD versus 10.4% UC, with arthropathy significantly more common in CD (12.9%) (100). Of note, altered intestinal bacterial diversity and dissemination of specific species have been postulated as a mucosal origin for arthritis (101–104). In addition, in a TNF-transgenic model of small intestinal CD and polyarthritis (TNF^ΔARE^), the temporal onset of arthritis correlates with microbial dysbiosis and the development of intestinal TLT (34, 50, 52). Thus there is precedent that intestinal TLT may aberrantly develop antibodies on a background of failed immunoregulation and thus integrate into a mechanism of systemic immune dysregulation.

## What are the Antigenic Stimuli Driving the Development of Intestinal B Cell Follicles in TLT?

There are several mechanisms that may underlie the increased numbers of ectopic B-cell follicles in the intestine of patients with IBD. This may be the result of non-specific polyclonal proliferation of B-cells, responding to the local production of B-cell activating factors such as cytokines (IL-6, IL-21, LTβ, and TNFα) in the inflamed gut. Alternatively and more likely, follicular B-cells within intestinal TLT may indicate a specific humoral immune reaction. There remains limited data on the (oligo) clonality of B cells from IBD patients during active disease or in preclinical models to address this. In addition, it is presently unclear against which antigen(s) are intestinal B-cells proliferating. Presumably both microbial and auto-antigens [including degradation products from extracellular matrix (ECM)] should be considered in the setting of TLT during intestinal immune dysregulation (see Figure 1).

### Intestinal Dysbiosis and Chronic Bacterial Infection

The increase in lymphocytes in the intestine during IBD and their organization into ectopic B cell lymphoid follicles are consistent with an orchestrated adaptive immune response, which is believed to develop in relation to chronic microbial colonization. Several observations favor this hypothesis, including a temporal correlation between the development of small intestinal TLT and a marked dysbiosis of the commensal microbiome (34, 52, 59) (see Figure 1). Alterations of the commensal flora are now considered a feature of human IBD, and our understanding of the profound effects that has on intestinal immune homeostasis is rudimentary [reviewed in Ref. (105)]. Patients with IBD respond favorably to antibiotics and fecal diversion, and have greater antibody titers against indigenous bacteria than unaffected individuals (105, 106). Inflammatory lesions are more pronounced in areas of the intestine that contain the greatest number of bacteria. The data in animal models provide further evidence for the involvement of gut bacteria in IBD. Pre-treatment with antibiotics has been shown to alleviate intestinal inflammation in several animal models (107).

### Extracellular Matrix Products and Molecular Mimicry

A specific immune response against self-antigens present in intestinal tissue could also be the initiating trigger for ectopic TLT generation. In the intestine of patients with IBD, there is a chronic inflammatory response present with the active recruitment of inflammatory cells and concomitant tissue damage. A resulting immune response can then be directed against intestinal matrix proteins, which can be recognized as neo-antigens. The ECM, composed of proteoglycans (including hyaluronan), collagens, elastin, and non-collagenous glycoproteins in turn both regulates and adapts to this inflammatory milieu. In fact, accumulation of ECM products has been shown to activate and recruit immune cells like T cells and monocytes during IBD (108). Proteolytic degradation of ECM components is a pathognomonic feature of a multitude of inflammatory and degenerative diseases [reviewed in Ref. (109)] and is mainly under the control of specific disintegrins and metalloproteinases (110). In addition, products of infectious agents, e.g., heat shock proteins and enzymes responsible for citrullination, have been shown in several models to induce immune reactivity. For example, several citrullinated autoantigens can be identified in tests for anti-citrullinated peptide antibodies (ACPA; Anti-cyclic Citrullinated Peptide; Anti-CCP), keratin, fibrinogen, fibronectin (FN), collagen, and vimentin from patients with arthritis (111, 112). It is plausible that infiltrating B cells, attracted by the TLT chemokine gradients, are educated against “self” proteins/immune complexes and start producing antibodies against the ECM degradation products. This has been demonstrated in both the joints of patients wit arthritis and in the lungs of patients with emphysema (both sites for TLT development) (112–114).

## Summary

It has been estimated that the intestine-associated GALT constitute approximately 50% of our immune cells and both the prenatally defined MLN and PP along with the postnatal induced CP and ILF maintain a remarkably tolerant environment. A staggering reality of IBDs is that the control of the collective regional immune system fails with dire consequences for the host. The florid appearance of TLT within the chronically inflamed intestine may indicate an attempt to support the failed immunoregulatory pathways and restore control of dysregulated inflammation. There remains a dearth of knowledge on the biological role played by TLT utilizing chronic models of IBD in addition to limited human data. Understanding how TLT integrate into the pathophysiology of IBD remains a critical question in our understanding of intestinal immunity.

## Future Directions and Knowledge Gaps

Since its discovery in 1932, the earliest histopathologic features of CD have included the extensive TLT formation within the inflamed mucosa. However, over 80 years later, our understanding of the role(s) of intestinal TLT in CD remains elusive and a crucial need for empirical evidence as to their function remains. The most pertinent question remains as to whether TLT are driving dysregulated immune pathology (e.g., autoantibodies or naive T cell education) or whether they trigger exuberant immune responses at sites of bacterial invasion or neo-antigen exposure (e.g., generation of IgA). Recent work has started to investigate similar questions in the setting of mesenteric fat-associated tertiary lymphoid follicles and has elegantly demonstrated for the first time somatic hypermutation and IgG generation *in situ* within TLT (115). Surprisingly, an exhaustive cellular profile of TLTs in human CD tissues has not been performed. As such, basic questions as to the cellular make up and as such, immune profile of TLT during active inflammation in CD is limited (e.g., do they produce IgA or IgG?). In addition, how TLT respond to current therapeutic interventions during CD is limited, and hampered by the inability to assess their presence or response to treatments *via* endoscopic or histologic means (e.g., with limited tissue from pinch biopsies).

A therapeutic gap also remains for the site-directed targeting of TLT structures while sparing lymph node physiology and the ability to rapidly respond to infections (e.g., either to selectively induce intestinal ILF to increase IgA and antimicrobial defense or to deplete ectopic TLT in situations where they generate autoantibodies and aberrantly activate naive T cells). For example, while a plethora of studies have utilized Lymphotoxin-β receptor blockade to disaggregate mature TLTs, few report on the subsequent impact on disease pathology. In addition, while LTβR inhibition will inhibit clustering of TLT in almost all preclinical models of disease, it has profound effects on lymph node and splenic architecture, e.g., loss of marginal zone macrophage and B cells (116), disruption of GCs, HEV repression (117), and altered DC ratios (118). While these experiments serve as proof-of-principle studies, the site-directed delivery of therapeutics that target TLT function may prove a more viable modality for the treatment of chronic inflammatory diseases, with limited effects on systemic anti-microbial immunity.

An improved understanding of TLT development and function will shed light on critical questions pertaining to intestinal immunity and host defense, and future detailed investigations into the functional role of TLT in intestinal immune dysregulation are sure to expand our understanding of the pathogenesis of CD.

## Author Contributions

EM and JR-N decided on the emphasis and outlined the article. EM wrote the main body of the piece. JR-N edited the piece.

## Conflict of Interest Statement

The authors declare that they are absence of any commercial or financial relationships that could be construed as a potential conflict of interest.

## Abbreviations

AID, activation-induced deaminase; BALT, bronchus associated lymphoid tissue; CD, Crohn’s disease; FDC, follicular dendritic cell; GALT, gut-associated lymphoid tissue; GC, germinal center; HEV, high endothelial venule; IBD, inflammatory bowel disease; ILC, innate lymphoid cell; ILF, isolated lymphoid follicle, SLO, secondary lymph node; LP, lamina propria; LTβR, lymphotoxin beta receptor; LTi, lymphoid tissue inducer cells; MLN, mesenteric lymph node; MMP, matrix metalloprotease; NOD1, nucleotide-binding oligomerization domain containing 1; RORγ, ROR-related orphan receptor gamma; SFB, segmented filamentous bacterium; Tfh, follicular helper T cells; TLT, tertiary lymphoid tissue; UC, ulcerative colitis.
